# Eisenmenger syndrome with left main compression syndrome: a case report

**DOI:** 10.1186/s12872-022-02524-w

**Published:** 2022-03-05

**Authors:** Charlotte Johanna Cool, Fathy Fathini, Ibnu Adams, Aninka Saboe, Nuraini Yasmin Kusumawardhani, Astri Astuti, Achmad Fauzi Yahya

**Affiliations:** 1 Congenital Heart Disease Division, Department of Cardiology and Vascular Medicine, University of Padjadjaran, Hasan Sadikin General Hospital, Jl. Pasteur No. 38, Bandung, 40161 Indonesia; 2 Department of Cardiology and Vascular Medicine, University of Padjadjaran, Hasan Sadikin General Hospital, Bandung, Indonesia; 3 Cardiology Intervention Division, Department of Cardiology and Vascular Medicine, University of Padjadjaran, Hasan Sadikin General Hospital, Bandung, Indonesia; 4 Cardiovascular Imaging Division, Department of Cardiology and Vascular Medicine, University of Padjadjaran, Hasan Sadikin General Hospital, Bandung, Indonesia

**Keywords:** Pulmonary hypertension, Eisenmenger syndrome, Left main coronary artery disease, Coronary artery compression

## Abstract

**Background:**

Left main coronary artery disease secondary to pulmonary artery compression related to Eisenmenger syndrome is an under-suspected condition that can cause fatal outcomes if left untreated. It presents with typical angina but is frequently mistaken for pulmonary hypertension (PH) symptoms. It is now recognized as one of the few important causes of angina in PH.

**Case presentation:**

A 37-year-old man with a history of unoperated atrial septal defect and Eisenmenger syndrome came to the outpatient department with a chief complaint of angina on exertion. Electrocardiogram showed regular sinus rhythm with right axis deviation, right ventricular hypertrophy, deep T-wave inversion in inferior and anterior leads suggestive of ischemia or strain, and incomplete right bundle branch block. Cardiac CT showed compression of the left main coronary artery due to a dilated main pulmonary artery. Therefore, this patient was diagnosed with Eisenmenger syndrome with left main compression due to dilated pulmonary artery. He was treated successfully with IVUS-guided stent implantation. The patient experienced marked improvement in regular activities, with no recurrence of angina symptoms. Angiography 3 months after the procedure revealed good patency of the stent, without significant stenosis.

**Conclusions:**

Left main coronary artery compression is a complication that should be suspected in patients with Eisenmenger syndrome presenting with angina symptoms. Non-invasive modalities are recommended for diagnostic evaluation, but the gold-standard technique remains coronary angiography. The best treatment is not well-established, with either myocardial revascularization or PH treatment, but a left main coronary artery stenting procedure is considered an ideal emergent treatment to provide a better quality of life for patients in this condition.

## Background

Eisenmenger syndrome is the most severe form of pulmonary hypertension (PH) in congenital heart disease (CHD). Patients with Eisenmenger syndrome generally present with typical signs such as cyanosis followed by dyspnoea or angina on exertion. Angina in PH has various aetiologies, such as mitral valve disease, disorders of the lungs, pulmonary artery (PA) disorders and congenital malformations. However, angina is also a common manifestation of a mechanical complication in PH: left main coronary artery (LMCA) compression. This condition increases the risk of sudden death due to myocardial territory at risk, and therefore may worsen the prognosis. It is usually an under-suspected and under-reported cause of angina, but the incidence is high, ranging from 19 to 44% [1]. Early recognition is the key to early management to improve patient outcomes.

Here we describe a case report of an unoperated atrial septal defect (ASD) patient with Eisenmenger syndrome who experienced LMCA compression due to a dilated main PA. This report will highlight the diagnostic workup and best possible management.

## Case presentation

**Fig. 1:**
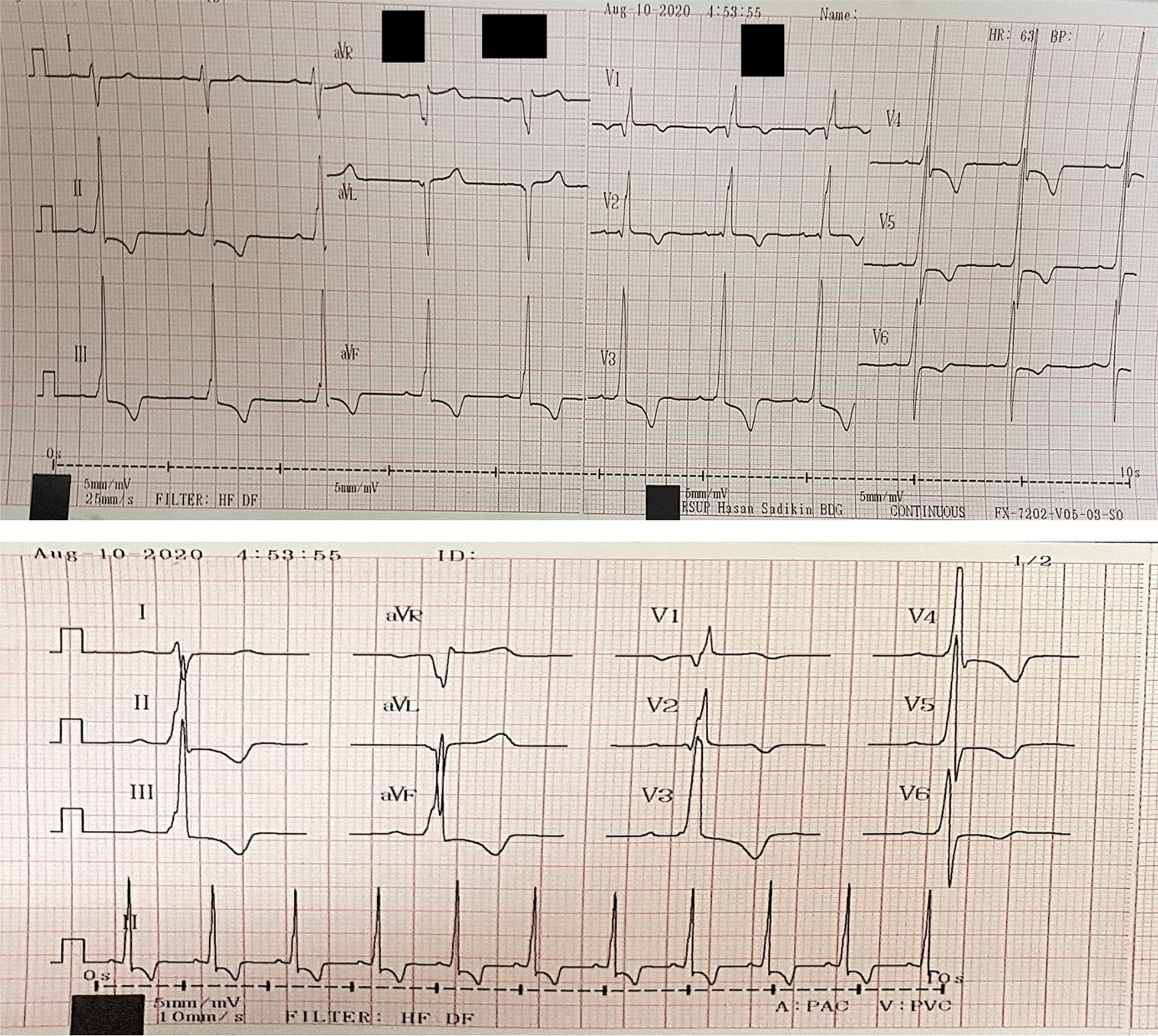
ECG showed regular sinus rhythm with RAD, RVH, deep T-wave inversion in inferior and anterior leads suggestive of ischemia or strain, and incomplete RBBB

**Fig. 2:**
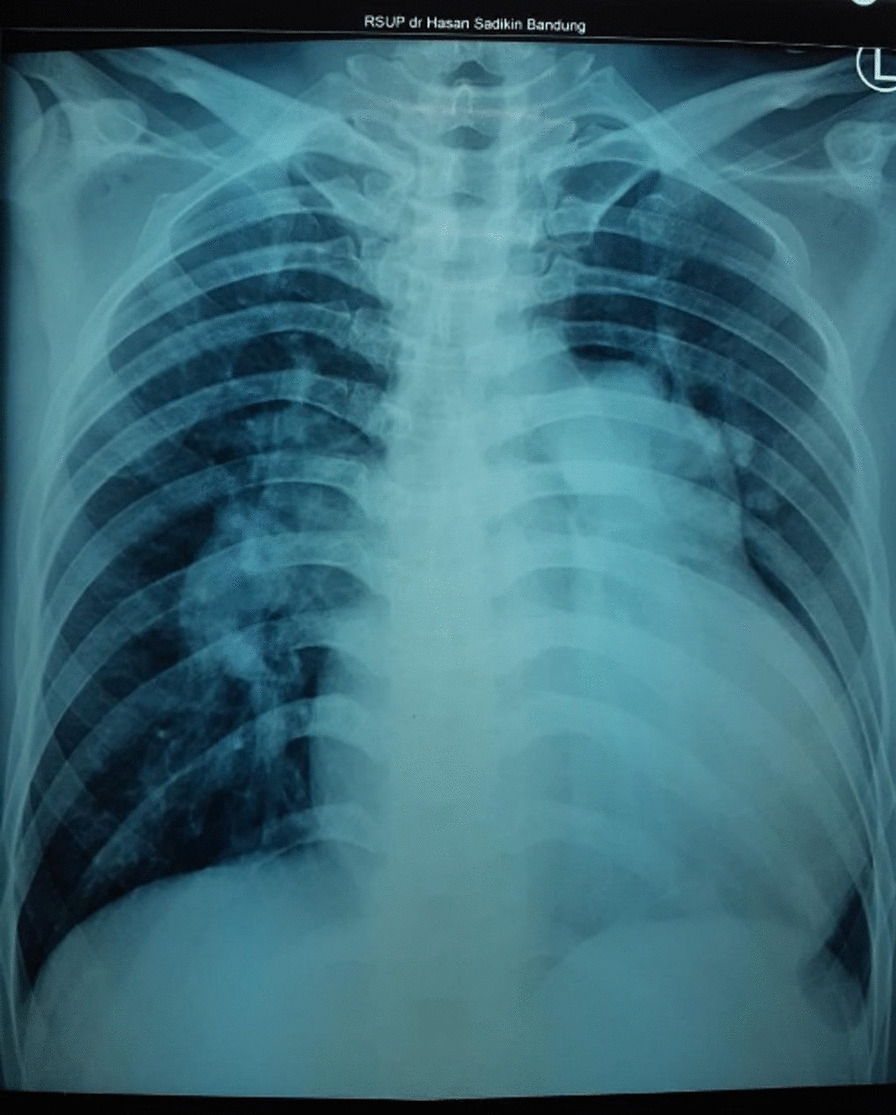
Thorax X-ray showed cardiomegaly with prominent pulmonary conus and increased pulmonary vascularity

**Fig. 3:**
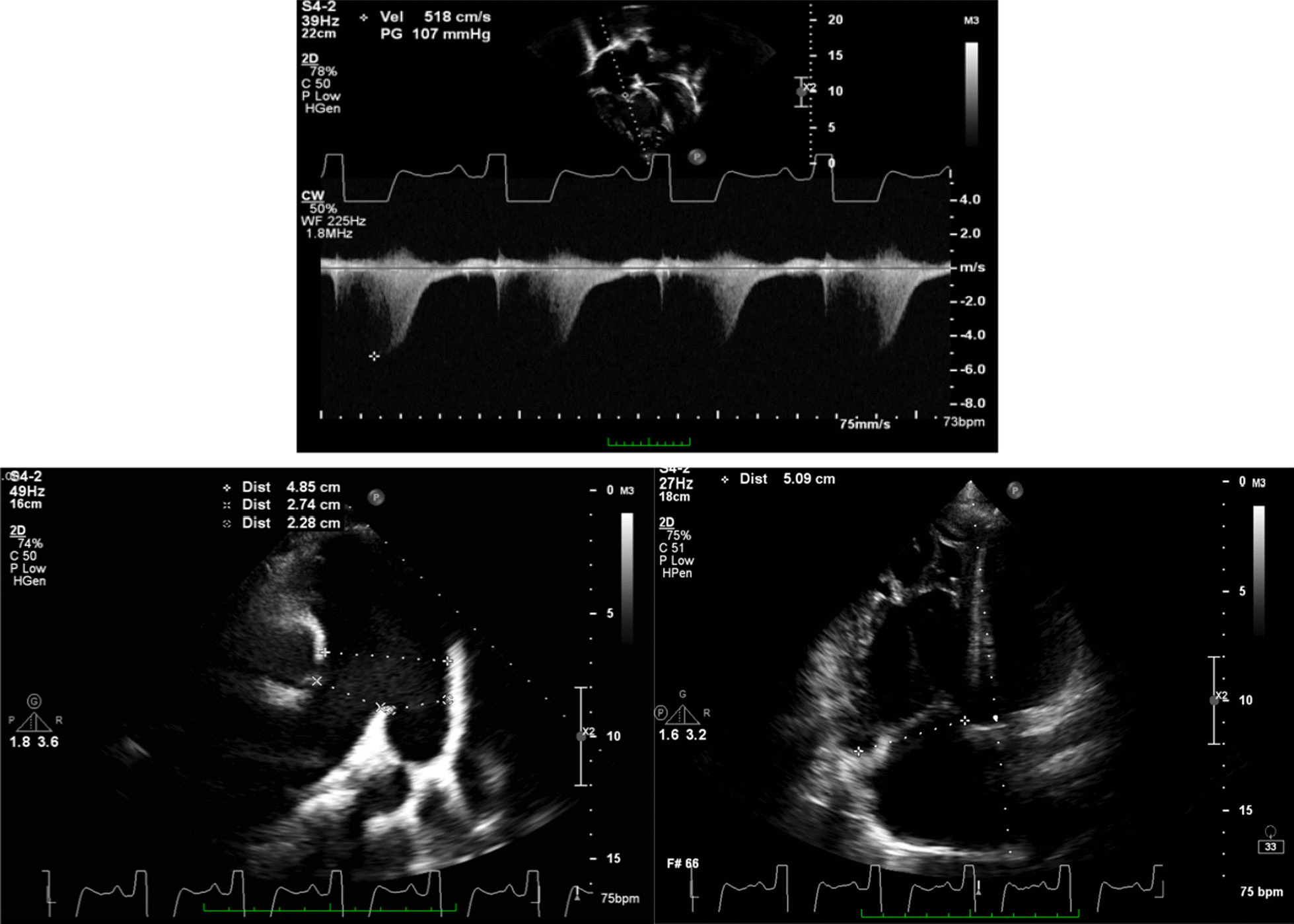
Transthoracic echocardiography images showed high probability of PH, (upper) with peak TR velocity of 5.1 m/s, (lower left) dilated MPA of 48 mm, (lower right) dilated RV of 51 mm

In this case report, we present a 37-year-old man with known unoperated secundum ASD and Eisenmenger syndrome. The patient was diagnosed for the first time 6 months before, with symptoms of typical angina on exertion that had worsened for the last 2 months. He was referred due to resource limitations from another hospital to the CHD division for follow-up in the cardiology department outpatient clinic and underwent scheduled examination. The patient had no other medical history, no family history of congenital disease and no prior invasive procedures. Physical examination showed a cyanotic appearance with finger clubbing, 86% oxygen saturation by peripheral pulse oximetry, signs of right heart congestion, cardiomegaly, accentuated P2, and pansystolic murmur at the lower left sternal border, with Carvallo’s sign on auscultation. The haematology parameters showed secondary erythrocytosis with a haemoglobin level of 19.5 g/dL and a haematocrit fraction of 55.5%. Electrocardiogram (ECG) showed a regular sinus rhythm with right axis deviation (RAD), right ventricular hypertrophy (RVH), deep T-wave inversion in the inferior and anterior lead suggestive of ischemia or strain, and incomplete right bundle branch block (RBBB; Fig. 1). Chest X-ray showed cardiomegaly with prominent pulmonary conus and increased pulmonary vascularity (Fig. 2). Transthoracic echocardiography (TTE) revealed secundum ASD with bidirectional shunt, mainly right to left (R to L) shunt, D-shaped left ventricle (LV), dilation of the right chamber (right atrium [RA] and right ventricle [RV]) and left atrium (LA), normal systolic function with paradoxical septal movement, moderate tricuspid regurgitation (TR), and a marked dilated main PA (diameter of 48 mm; Fig. 3). The TTE examination conclusion based on ESC guidelines for the diagnosis and treatment of PH was secundum ASD with a high probability of PH and marked main pulmonary artery (MPA) dilation.

**Fig. 4:**
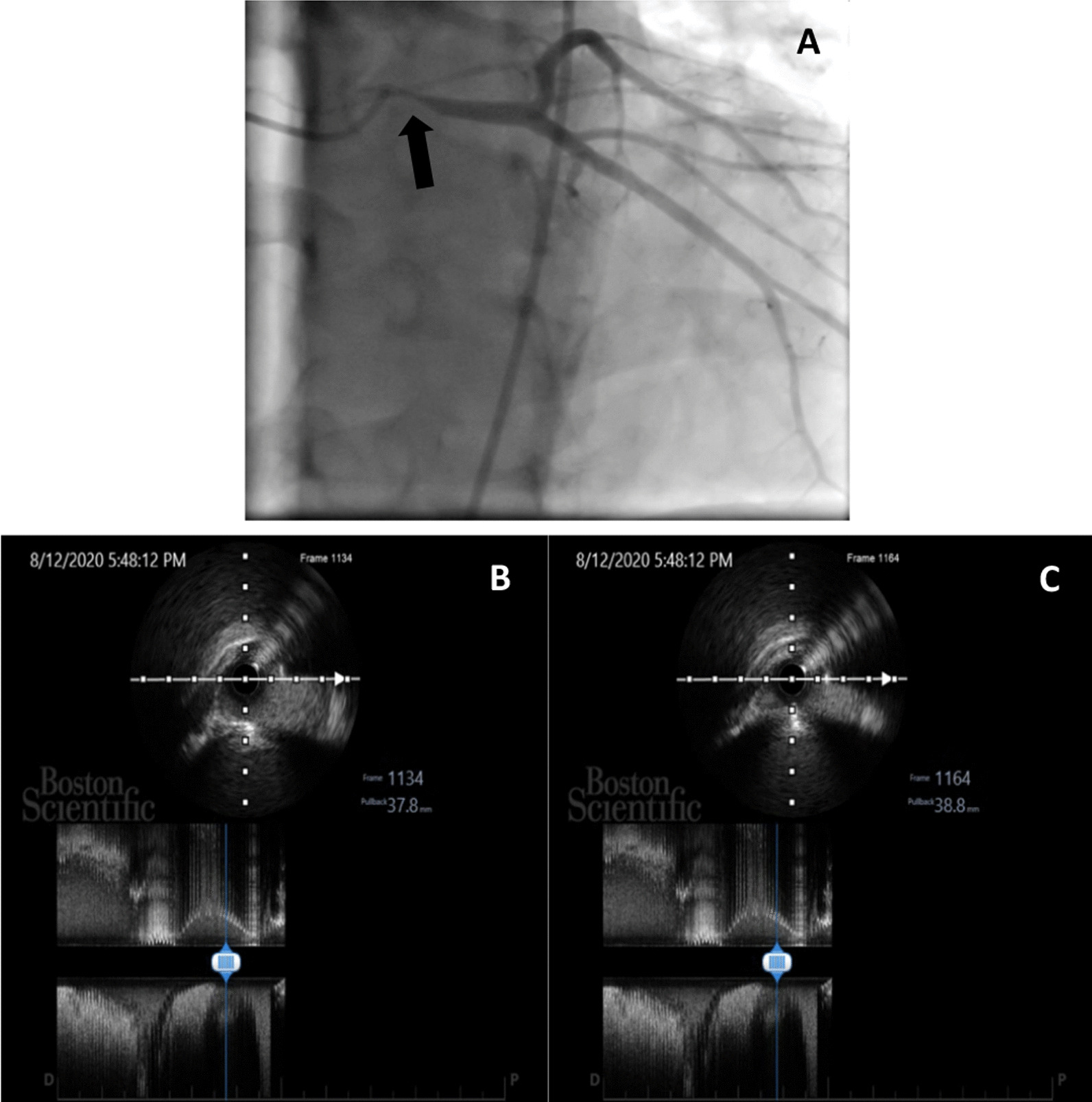
**A** Angiography showed severe LMCA stenosis in LAO-caudal view; **B**, **C** IVUS evaluation showed dynamic compression of LMCA

**Fig. 5:**
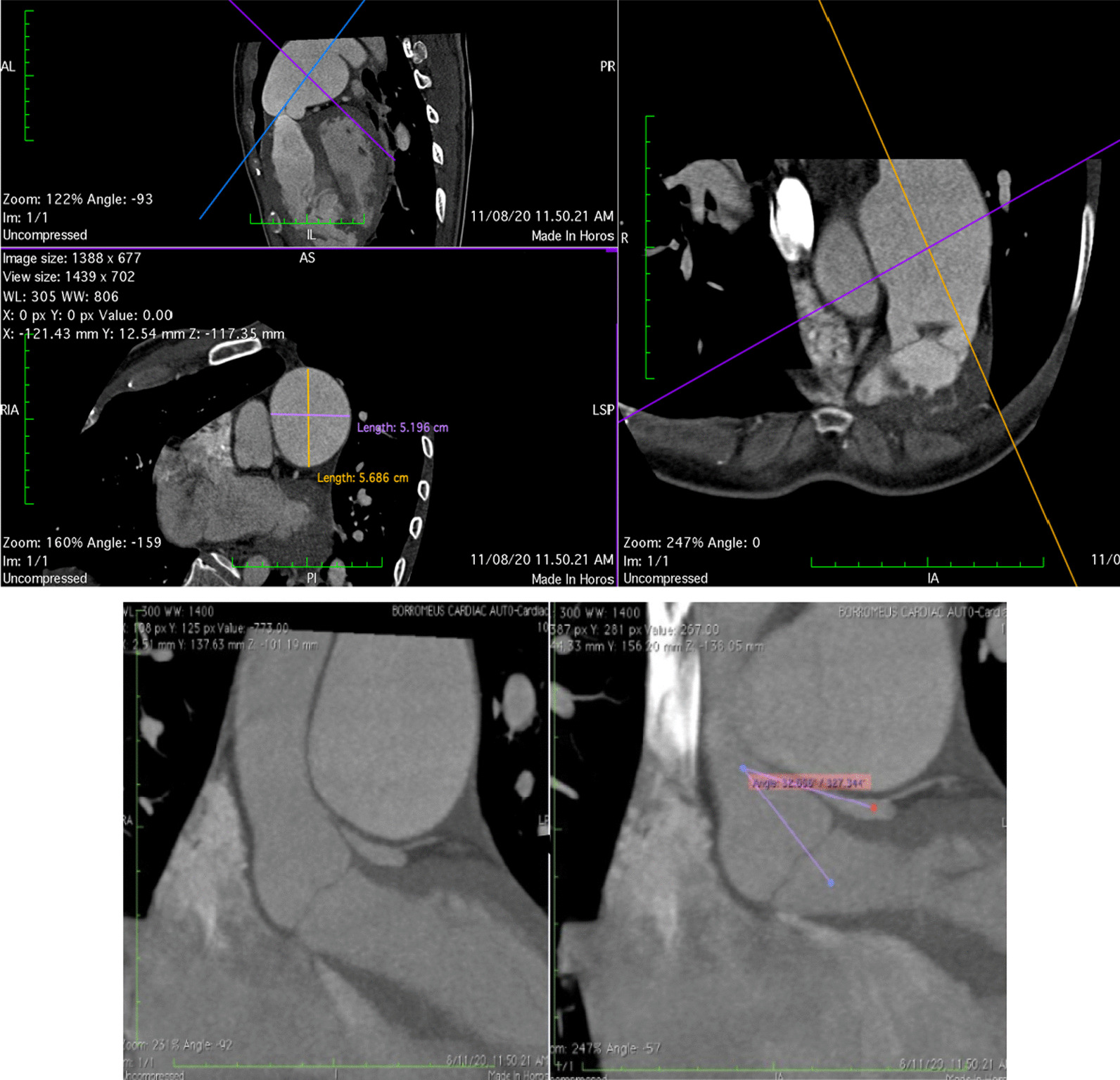
Contrast-enhanced cardiac CT images, (upper) images showed dilated MPA of 56.8 × 51.9 mm, (bottom left) black arrows showed severe compression of LMCA and (bottom right) LM take-off angle of 32°

Based on the presented data, the patient was scheduled for elective right heart catheterization (RHC), as well as angiography due to suspected ischemia suggesting a mechanical complication due to marked dilation of the PA. The RHC revealed secundum ASD with bidirectional (mainly R to L) shunt, mean pulmonary artery pressure (mPAp) pre–oxygen test of 78 mmHg decreasing to 70 mmHg after oxygen test, with low flow (flow ratio pre–oxygen test of 0.98 and post-oxygen test of 1.09) and high resistance (pulmonary vascular resistance pre–oxygen test of 35.3 decreasing to 31.1 Wood units after oxygen test), with a non-reactive oxygen test as the conclusion. Angiography (Fig. 4A) revealed severe stenosis at the ostium part of the LMCA, without significant stenosis at other coronary arteries. To confirm the diagnosis, the patient was then sent to the radiology department and underwent a contrast-enhanced computerized tomography (CT) scan. The cardiac CT (Fig. 5) showed a dilated MPA (56.8 × 51.9 mm in diameter), MPA-to-aorta ratio of > 1.5, left main (LM) take-off angle of 32° and severe LMCA compression. Based on coronary angiography and cardiac CT findings, the patient was then referred to a coronary interventionist for further assessment and intervention. Intravascular ultrasound (IVUS) evaluation showed dynamic compression in the ostium LM without atherosclerosis (Fig. 4B, C). After a thorough examination, the patient was diagnosed with LM compression syndrome due to dilated pulmonary artery caused by the severe form of PH Eisenmenger syndrome.

**Fig. 6:**
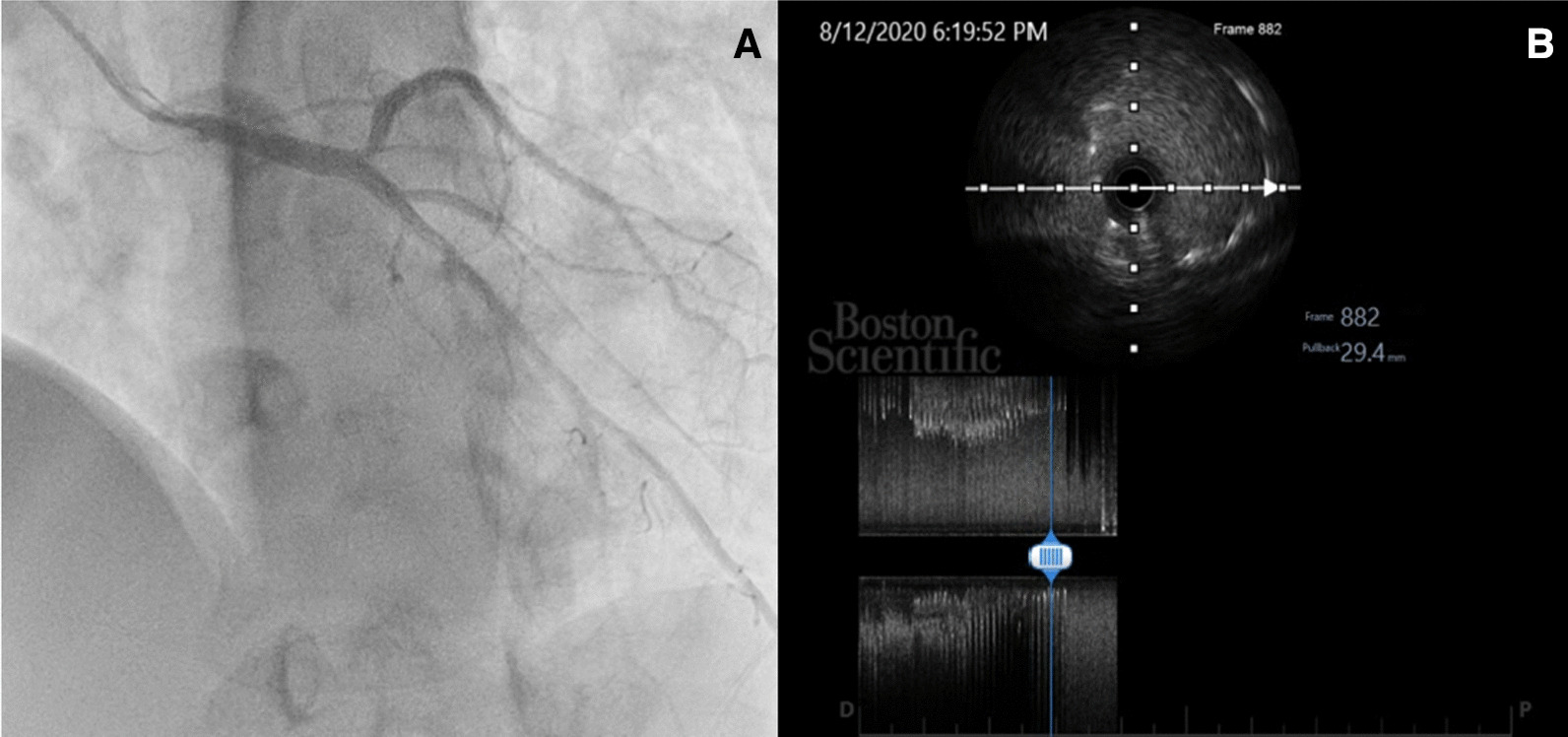
**A** Successful PCI of the LMCA in LAO-caudal view; **B** IVUS evaluation post-PCI of LM

Percutaneous coronary intervention (PCI) with a new-generation zotarolimus-eluting stent, size 4.5 × 22 mm, was successfully implanted with IVUS guidance, and the evaluation showed good stent placement (Fig. 6A, B). The patient was discharged after 7 days of hospitalization with no complication and given dual antiplatelet therapy (81 mg of acetylsalicylic acid and 75 mg of clopidogrel once daily). PH was treated with a combination of sildenafil, a potent and selective inhibitor of phosphodiesterase type 5; and beraprost, a prostacyclin analogue. On clinical follow-up a week and then a month after hospitalization, the patient showed no recurrence of angina symptoms and was scheduled for evaluation 3 months after the procedure. Angiography evaluation after 3 months revealed no significant stenosis and good patency of the stent. The patient underwent routine follow-up in the outpatient clinic of the CHD division, with marked improvement in regular activities and good compliance.

## Discussion and conclusions

Eisenmenger syndrome is a severe form of PH associated with acyanotic CHD [2, 3]. A persistent haemodynamic condition in chronic PH may further cause PA dilation due to increased PA pressure (PAp), based on Laplace’s law [1, 4]. PA dilation is present in around 76.6% of patients with severe PH [5]. Assessment using non-invasive imaging modalities is recommended to evaluate PH parameters, including PA diameter. TTE can measure the PA diameter at end-diastole from the parasternal short-axis view just before the bifurcation, with a normal value of less than 25 mm [1, 6]. In case of any difficulties in determining the value precisely, a contrast-enhanced CT scan is also suggested to identify PH parameters [5].

Angina symptoms in PH are important clinical features. The differential diagnosis of angina is extensive, and the characteristics of PH resemble angina pectoris in general and in location, radiation, intensity, quality and tendency to be initiated by physical exertion. The mechanism of angina is usually due to unmatched metabolic demands caused by reduced coronary perfusion and the pressure gradient of the RV and PA. This can be caused by different underlying diseases, such as mitral valve stenosis; lung disease, especially asthma or emphysema; pulmonary embolism; or CHD [7, 8]. In some cases, the angina presentation may be related to a mechanical complication of PH, and Table 1 lists some other common complications [3, 8, 9].

**Table 1:** Common complications of Eisenmenger syndrome. [3, 31, 32]

No	Complications	Signs and symptoms
1	Secondary erythrocytosis	Hyperviscosity symptoms
2	Haemostasis abnormalities (increased risk in both bleeding and thrombosis)	Pulmonary bleeding (rupture of hypertrophied bronchial arteries) Pulmonary embolism Deep vein thrombosis (DVT) Stroke or brain abscess
3	Arrhythmias	Ventricular arrhythmias (ventricular tachycardia [VT] or ventricular fibrillation [VF]) Supraventricular arrhythmias (atrial fibrillation [AF] or atrial flutter [Afl])
4	Endocarditis	Fever
5	Mechanical complications Compression of LMCA	Chest pain (angina-like or not)
6	PA aneurysms, rupture and dissection	Acute dyspnoea on exertion Haemodynamic decompensation Sudden death
7	Compression of intrathoracic structures (recurrent laryngeal nerves)	Hoarseness
8	Cerebral hypoperfusion due to low cardiac output	Syncope
9	Renal dysfunction	Reduced urine output Hyperuricaemia

Coronary artery disease (CAD) in PH patients presenting with angina is often under-suspected and usually diagnosed as a coincidence finding. It is now recognized as a serious mechanical complication of PH [2]. CAD pathophysiology is commonly secondary to atherosclerotic disease, but another known condition responsible is PH, which involves extrinsic compression of the LMCA due to dilation of the PA. Extrinsic compression occurs due to a closed anatomic relationship between two structures: the MPA and LMCA [10, 11]. The MPA serves as a short and wide pipe that normally travels along the left of the ascending aorta and bifurcates just below the aortic arch to form the right and left PA [12]. Among the adjacent structures are the coronary arteries, which originate from the aortic sinuses of the aorta and pass alongside the pulmonary trunk [13].

LMCA compression by an enlarged PA in patients with PH was first described in 1957 by Corday et al. [14]. The prevalence rate was around 40%, especially if accompanied by angina symptoms [15]. These cases were rarely reported, due to under-diagnosis despite its high incidence [10]. We find some similar conditions previously reported in case reports (Table 2). The mechanism of angina in this condition is still uncertain. Progressive increases in Pap and RV overload, along with myocardial ischemia caused by artery compression, are proposed as possible mechanisms [9].

**Table 2:** Literature review of case reports of LM compression due to dilated PA causing angina

Author/Year	Patient	Aetiology of PH	Diagnostic modalities	Management	Outcome/follow-up
Yusuke Jo, Akio Kawamura. 1987 [33]	Female, 42 years old. Atypical angina	Secundum ASD, PH	**MSCT:** MPA diameter 47 mm **Angiography:** significant LM stenosis	Surgical ASD closure	Resolution of stenosis and reduced PA diameter after 4 months
Diana Bonderman, Dominik Fleischmann. 2002 [34]	Female, 62 years old Atypical angina	CTEPH	**MSCT:** dilated MPA **Angiography:** ostial stenosis of LM	Pulmonary thromboendarterectomy	Size regression of PA and compression resolved, no further diagnostic follow-up
Susana Gomez Varela, Pedro M. Montes Orbe. 2004 [8]	Female, 31 years old. Atypical angina	Suspected primary PH	**MSCT:** dilated MPA **MRI:** dilated MPA, diameter 40 mm **Angiography:** 80% stenosis ostium LM	Continuous apoprotein infusion and PCI	Complete remission of symptoms, no diagnostic follow-up
Jonathan D. Dodd, Andrew Maree. 2007 [35]	Male, 28 years old. Atypical angina	PDA, Eisenmenger syndrome	**MSCT:** dilated MPA **Angiography:** severe LM stenosis	PCI	Follow-up 4 months after the procedure showed no recurrence of angina
Morteza Safi, Vahid Eslami. 2008 [36]	Female, 64 years old. Atypical angina	Sarcoidosis, CTEPH	**MSCT:** dilated MPA, RA thrombus	Embolectomy	N/A
Angel E. Caldera, Ignacio Cruz-Gonzalez. 2009 [37]	Female, 48 years old. Atypical angina	Post-surgical PDA, Eisenmenger syndrome	**MSCT:** MPA diameter 63 mm **Angiography:** severe ostial and proximal stenosis of LMCA	IVUS-guided PCI	Symptoms improvement and CT evaluation after 6 months revealed patent stent
Tomoharu Kawase, Hironori Ueda. 2010 [38]	Male, 43 years old. Typical angina	Group 3 PH	**MSCT:** dilated MPA **Angiography:** severe stenosis of LM	IVUS-guided PCI	Angiography evaluation after 3 months showed good stent position
Tobias Koppara, Julinda Mehilli. 2011 [2]	Female, 16 years old. Typical angina	Perimembranous VSD, Eisenmenger syndrome	**MRI:** dilated MPA **Angiography:** severe LM stenosis	PCI	Follow-up 6 months after the procedure showed no recurrence of angina, no further diagnostic follow-up
Carlo Pace Naopleone, Emanuela Angeli. 2012 [30]	Female, 45 years old. Typical angina	Sinus venosus ASD, PH	**MSCT:** MPA diameter 42 mm **Angiography:** isolated LM stenosis	Surgical correction (ASD closure, reduction plasty of pulmonary trunk)	CT evaluation confirmed relief of LM compression, follow-up 6 months after the procedure showed no recurrence of angina
Kristina Andjelkovic, Dimitra Kalinovska. 2013 [20]	Female, 37 years old. Dyspnoea	Primum ASD, Eisenmenger syndrome	**TTE:** MPA diameter 43 mm **Angiography:** significant LM stenosis	IVUS-guided PCI	No further diagnostic follow-up
Kothandam Sivakumar, Francis Gnanapragasam. 2014 [9]	Male, 58 years old. Typical angina	PDA, Eisenmenger syndrome	**TTE:** dilated MPA **Angiography:** stenosis of LM	PCI	CT evaluation after 6 months showed patent stent in LM
Luciana F. Seabra, Henrique B. 2015 [19]	Female, 39 years old. Typical angina	Idiopathic PAH	**MSCT:** MPA diameter 44 mm **Angiography:** LMCA critical obstruction	IVUS-guided PCI	No further diagnostic follow-up
K. Chernichka, N. Danilov. 2015 [39]	Female, 32 years old. Typical angina	Idiopathic PAH	**MSCT:** MPA diameter 47 mm **Angiography:** significant LM stenosis	Conservative with PH therapy	N/A
Eduardo Belisario Falchetto, Jamil Abdalla Saad. 2015 [40]	Male, 66 years old. Atypical angina	Schistosomiasis	**MSCT:** MPA diameter 80.4 mm **Angiography:** severe LM stenosis	IVUS-guided PCI	CT evaluation after 8 months showed well-positioned stent
Kadhem Albadri, Jesper M. Jensen. 2015 [41]	Female, 49 years old. Typical angina	Idiopathic PAH	**TTE:** MPA diameter 42 mm **MSCT:** dilated MPA **Angiography:** severe osteal LMCA stenosis	IVUS-guided PCI	Completely resolved symptoms, no further diagnostic follow-up
Ryutaro Ikegami, Kauzuyuki Ozaki. 2017 [42]	Female, 65 years old. Typical angina	ASD, Eisenmenger syndrome	**MSCT:** dilated MPA **Angiography: **ostium LM stenosis	IVUS-guided PCI	CT evaluation after 3 months confirmed stent patency. Angiography evaluation after 6 months showed neither compression nor restenosis
Lara Teixeira de Araujo, Pammela Jacomeli Lembi. 2018 [43]	Male, 54 years old Typical angina	Portopulmonary hypertension (POPH) secondary to alcoholic liver cirrhosis	**MSCT:** dilated MPA **Angiography:** severe osteal LM stenosis	PCI	Significant improvement in symptoms and functional capacity, no further diagnostic follow-up
Ibrahim Basarici. 2020 [44]	Female, 39 years old. Atypical angina	PDA, PH	**MSCT:** dilated MPA **Angiography:** complete LM stenosis	Scheduled for CABG and PA aneurysm repair, but refused and only given PH therapy	N/A

Diagnostic modalities regarding this condition are fundamental. Contrast-enhanced CT should be performed to define the PH parameters, especially MPA dilation, and define the LM compression [1, 15, 16]. The compression risk is related to some strong predictors measured by contrast-enhanced CT, such as PA diameter > 40 mm, ratio of the MPA and aortic root ≥ 1.5, and take-off angle < 45° (formed between the longitudinal line of the LMCA and orthogonal line of the aortic valve) [15, 17]. It is also determined by the anatomic relationship between the PA and the origin of the LMCA [18]. The accuracy of those predictors is somewhat favourable: the sensitivity and specificity for LM compression due to PA dilation > 40 mm are 83% and 70%, respectively. The MPA-to-aorta ratio has a sensitivity of 73% and a specificity of 70%, respectively, for predicting compression [15]. Nevertheless, the gold standard of modalities to confirm LM stenosis remains coronary angiography, particularly guided by IVUS [16, 19]. In our case, we found all the strong predictors for compression risk, such as marked dilation of the main PA (diameter 54 mm), MPA-to-aorta ratio > 1.5, and LM take-off angle 32°. All parameters were measured from cardiac CT, which also confirmed severe LMCA compression.

The best management for LMCA compression by PA dilation is not well-established due to the small number of cases reported, but emergent treatment is required due to the high mortality risk. Treatment of the PH itself and coronary revascularization is the optimal choice [9, 11]. However, the rapid improvement of PA dilation after adequate management is impossible; therefore, an urgent approach is needed to improve survival. Recent evidence shows that coronary stenting is a possible option to resolve LM compression [12, 24, 25]. An isolated lesion involving the ostium or shaft of the LM is a class II recommendation for PCI, whereas more complex lesions are best treated with a surgical bypass procedure. Coronary artery bypass graft (CABG) also involves a higher risk in general anaesthesia and cardiopulmonary bypass (CPB) in patients with underlying PH, so PCI is the preferred strategy. A drug-eluting stent (DES) is preferred compared to a bare-metal stent (BMS) because it is proven to improve survival, particularly guided by IVUS. It is also associated with fewer adverse cardiovascular events and a low restenosis risk in the atherosclerosis population;^21,22^ further studies are needed to confirm its application in the LMCA compression due to PH population. The choice of a newer-generation DES containing zotarolimus was based on the safety and effectiveness of the individualized approach to shortened dual anti-platelet duration in selected patients undergoing PCI, such as in high bleeding-risk patients, but this also needs further studies to assure safety in the PH population [23, 24]. The use of IVUS in this condition is to determine the characteristics of compression (whether there is an atherosclerotic plaque), precise part of involvement, ischemia burden of the stenosis estimation, stent optimization, and adequate expansion and apposition of the stent after LM PCI [21, 25].

The cornerstone of management is to manage the aetiology, which is the CHD. Defect closure is recommended to hopefully improve both PAp and LMCA compression, but further studies are needed to confirm this hypothesis [25]. Mainstay treatment of Eisenmenger syndrome has evolved in the past decade and is divided into several steps, including supportive therapy with oral anticoagulants, diuretics or oxygen therapy, with referral to PH centres to perform vasoreactivity testing to assess suitability for surgical repair [26, 27]. The use of PH targeted therapy such as endothelin receptor antagonists (ERA), phosphodiesterase type 5 (PDE-5) inhibitors, and prostacyclin analogues as monotherapy or drug combination are class I recommendations in pulmonary arterial hypertension (PAH) therapy according to the WHO functional classes stated in the ESC guidelines for the diagnosis and treatment of PH. Those drugs reduce the pressure elevation and diameter of the PA and are proven to improve angina symptoms. Invasive management such as balloon atrial septostomy (BAS) and lung transplantation are also considered after inadequate response to optimal medical treatment [3].

A surgical approach with aneurysmorrhaphy to reduce the PA diameter, aneurysmectomy to repair or replace the artery, and double-lung or heart–lung transplantation is recommended in PA aneurysm with compression of adjacent structures, although it carries a very high surgical risk [28, 29]. Novel PA reduction plasty is also the current preferred choice. The techniques include internal plication and outer layer reinforcement to hopefully reduce pressure, although further studies are necessary to establish this recommendation [29, 30].

LMCA compression is a complication that should be suspected in patients with Eisenmenger syndrome presenting with angina symptoms. Non-invasive modalities such as contrast-enhanced CT are recommended to detect both PH features and coronary artery compression, with acceptable accuracy. Invasive modalities also play an important role in confirming the diagnosis and as ideal management. An LM stenting procedure is considered the best therapeutic approach to improve long-term patient outcomes.

### Strengths

We successfully diagnosed and treated a patient with LM compression due to PA dilation in Eisenmenger syndrome, with a good outcome.

### Limitations

We present only one patient in this case report, and no specific guidelines exist for management. We also have limited resources, such as PH drugs, which are unavailable in our country, and human resources specializing in surgical procedures to optimize the management.

### Learning points

In Eisenmenger syndrome patients presenting with angina, mechanical complications such as LMCA compression should be considered as an aetiology. Proper evaluation and management are essential for a better prognosis.

## Data Availability

Not applicable.

## Abbreviations

PH: Pulmonary hypertension
PAH: Pulmonary arterial hypertension
CHD: Congenital heart disease
PA: Pulmonary artery
ASD: Atrial septal defect
ECG: Electrocardiogram
RAD: Right axis deviation
RVH: Right ventricular hypertrophy
RBBB: Right bundle branch block
TTE: Transthoracic echocardiography
LV: Left ventricle
RA: Right atrium
RV: Right ventricle
LA: Left atrium
TR: Tricuspid velocity
MPA: Main pulmonary artery
PAp: Pulmonary artery pressure
RHC: Right heart catheterization
PVR: Pulmonary vascular resistance
mPAp: Mean pulmonary artery pressure
LM: Left main
LMCA: Left main coronary artery
CT: Computerized tomography
IVUS: Intravascular ultrasound
CAD: Coronary artery disease
CPB: Cardiopulmonary bypass
CABG: Coronary artery bypass graft
DES: Drug-eluting stent
BMS: Bare-metal stent
ERA: Endothelin receptor antagonists
PDE-5: Phosphodiesterase type 5
BAS: Balloon atrial septostomy
